# Numerical Validation of mSOUND Using Fully Heterogeneous Skull Models

**DOI:** 10.1109/ojuffc.2026.3677158

**Published:** 2026-03-24

**Authors:** JEFFREY BELL, LU XU, HONG CHEN, YUN JING

**Affiliations:** 1 Graduate Program in Acoustics, Penn State University, State College, PA 16802 USA; 2 Department of Biomedical Engineering, Washington University in St. Louis, St. Louis, MO 63130 USA; 3 Department of Biomedical Engineering and Neurosurgery, Washington University in St. Louis, St. Louis, MO 63130 USA

**Keywords:** mSOUND, transcranial ultrasound, focused ultrasound, wave propagation

## Abstract

In this work, we present a wave solver incorporated into mSOUND, an open-source MATLAB toolbox for acoustic simulations. The solver achieves an accuracy-computational efficiency balance for acoustic simulations through strongly heterogeneous media. We demonstrate its usefulness in simulating transcranial focused ultrasound through fully heterogeneous skull models, in which each voxel is assigned a unique speed of sound, density, and absorption coefficient. We compare the solver’s computation times and accuracy against the widely-used full-wave solver k-Wave, achieving computational savings by two orders of magnitude while maintaining comparable accuracy, particularly in the focal region. To obtain a fully heterogeneous skull model, conversions from Hounsfield unit (HU) maps obtained via computed tomography (CT) scans to acoustic variables are needed. These conversions require a maximum speed of sound parameter to be assigned as a known input. In this work, we also investigate the effect changing this parameter has on the acoustic field as well as mSOUND’s accuracy relative to k-Wave. Over four skull samples and three maximum speeds of sound, our results indicate a decrease in focal pressure and increase in error for mSOUND in the focal region as the maximum speed of sound increases.

## I. INTRODUCTION

Focused ultrasound (FUS) has seen a number of new applications and innovations in recent years [1]. In particular, transcranial ultrasound (tFUS) has progressed as a non-invasive treatment modality in the brain due to imaging advancements such as magnetic resonance (MR) thermometry and passive cavitation mapping. These imaging techniques, coupled with high-powered phased arrays, allow for overcoming the difficulties induced by complex skull geometries via phase correction and active monitoring of the tFUS procedure. As a result, tFUS has been implemented to achieve thermal ablation, creating a lesion to disrupt pathological brain circuits in the thalamus associated with essential tremor [2], [3] and tremor-dominant Parkinson’s disease [4], [5]. Furthermore, tFUS has been shown to temporarily open the blood-brain barrier (BBB) to allow for targeted drug delivery to the brain for conditions including Alzheimer’s disease [6] and high-grade glioma [7]. In addition to these applications, tFUS is being explored as a non-invasive approach to neuromodulation [8], sonodynamic therapy [9], and the removal of intracranial hemorrhages [10].

Numerical simulations are a critical component in the development of these innovations as well as in the treatment planning process. Specifically, simulating wave propagation through the skull allows researchers and clinicians to optimize FUS parameters and predict thermal doses and the likelihood of cavitation in the brain [11], [12], [13]. Due to the high degree of precision needed for predicting the focal position, negative pressure component (for predicting cavitation), and thermal dose resulting from tFUS, high-fidelity models are necessary. At the same time, computational resources are not limitless for many users, and some applications are time-sensitive or iterative in nature [14], [15], necessitating a balance between accuracy and computational efficiency.

Acoustic wave solvers developed in the last 20 years for modeling tFUS generally have tended toward one of these competing desires. For instance, the most computationally efficient solvers can only accommodate homogeneous or simple layered media. Recent examples of this class include ray tracing-based solvers, some of which have also demonstrated comparable accuracy to other solvers [16], [17]. Meanwhile, many of the most accurate operate in the time domain, adding a temporal dimension to simulations which greatly increase their computational demands, especially since most tFUS applications are monofrequency. One such finite-difference time domain (FDTD) solver is the ‘Fullwave’ software which solves the full wave equation, accounting for quadratic nonlinearity, multiple relaxations, and propagation in heterogeneous media [18].

In this paper, we report a numerical method implemented in mSOUND, an open-source toolbox written in MATLAB, based on the extended split-step Fourier (ESSF) approach that accommodates wave propagation in arbitrarily heterogeneous media. More importantly, mSOUND is validated against k-Wave [19] for fully heterogeneous 3D skull models. Although mSOUND was included as one of the solvers in a recent intercomparison benchmark study [20], it was only applied to simplified skull models that lack full heterogeneity. Additionally, the algorithm implemented in the current version of mSOUND has not been reported to date and differs significantly from that of the original version [21].

ESSF was first introduced in 1992 by Walter Kessinger within the field of geophysics to handle severe lateral changes in the speed of sound [22]. This method was adopted and extended by mSOUND for 3D simulations of tFUS such that each voxel can be assigned a unique speed of sound, density, and absorption coefficient. High-resolution skull maps of the necessary acoustic properties are obtained via transformations from computed tomography (CT) scans of human subjects. There is a lack of consensus within the literature as to how to convert from the Hounsfield units (HU) obtained from CT scans to the acoustic properties [12], [14], [23], [24], [25], [26], [27]. Most incorporate some variation of a linear interpolation from HU to speed of sound, for instance. There is an additional complication: the maximum speed of sound used in the conversion must be specified, with sample reported values including 2900 m/s [23], 3100 m/s [24], 3507 m/s [25], and 4000 m/s [27]. As such, we will also report on an investigation into the effect of changing the maximum speed of sound on mSOUND’s accuracy compared to k-Wave. This work demonstrates mSOUND’s substantial computational savings while maintaining comparable accuracy relative to k-Wave for the more clinically relevant fully heterogeneous skull model.

The outline of the paper is as follows: section II details the theoretical framework for mSOUND’s acoustic wave solver; section III is focused on the approach and setup to compare mSOUND and k-Wave; section IV presents the results of the comparison and subsequent statistical studies; section V entails analysis of the results and possible explanations for the discrepancies between the solvers; section VI concludes the study and highlights future work.

## II. THEORY

We begin with the homogeneous Helmholtz equation:

(1) $$\nabla^{2}\text{p}+k^{2}\text{p}=0$$

where p is the pressure, assuming time-harmonic behavior and over the (x, y, z) domain, k=ω/c is the acoustic wavenumber ω is the angular frequency, and c is the speed of sound. A common and computationally efficient approach to solve this equation is the angular spectrum approach (ASA). If we assume one-way propagation in the +z-direction and take the 2D Fourier transform of p with respect to x and y, i.e., $P\left(k_{x},k_{y},z\right)=ℱ\{p(x,y,z)\}$, then (1) takes the form of an ordinary differential equation (ODE):

(2) $$\frac{\partial^{2}P}{\partial z^{2}}+k_{z}^{2}P=0$$

where $k_{z}=\sqrt{{\left(\frac{\omega }{c}\right)}^{2}-k_{x}^{2}-k_{y}^{2}}$ is the z-component of the wavevector. Then, the solution to (2) takes the form

(3) $$P=P_{0}e^{-jk_{z}z}$$

where $P_{0}$ is known as a boundary condition over the z=0 plane. Then, the acoustic pressure over Cartesian coordinates can be reobtained following an inverse Fourier transform of (3). For harmonic signals known over a source plane, ASA offers an extremely efficient method for determining the pressure distribution over any destination plane, owing to the use of the fast Fourier transform (FFT). For tFUS, extensions are needed to account for acoustic absorption, multiple internal reflections within the skull, and heterogeneities in the speed of sound and density.

**FIGURE 1.**
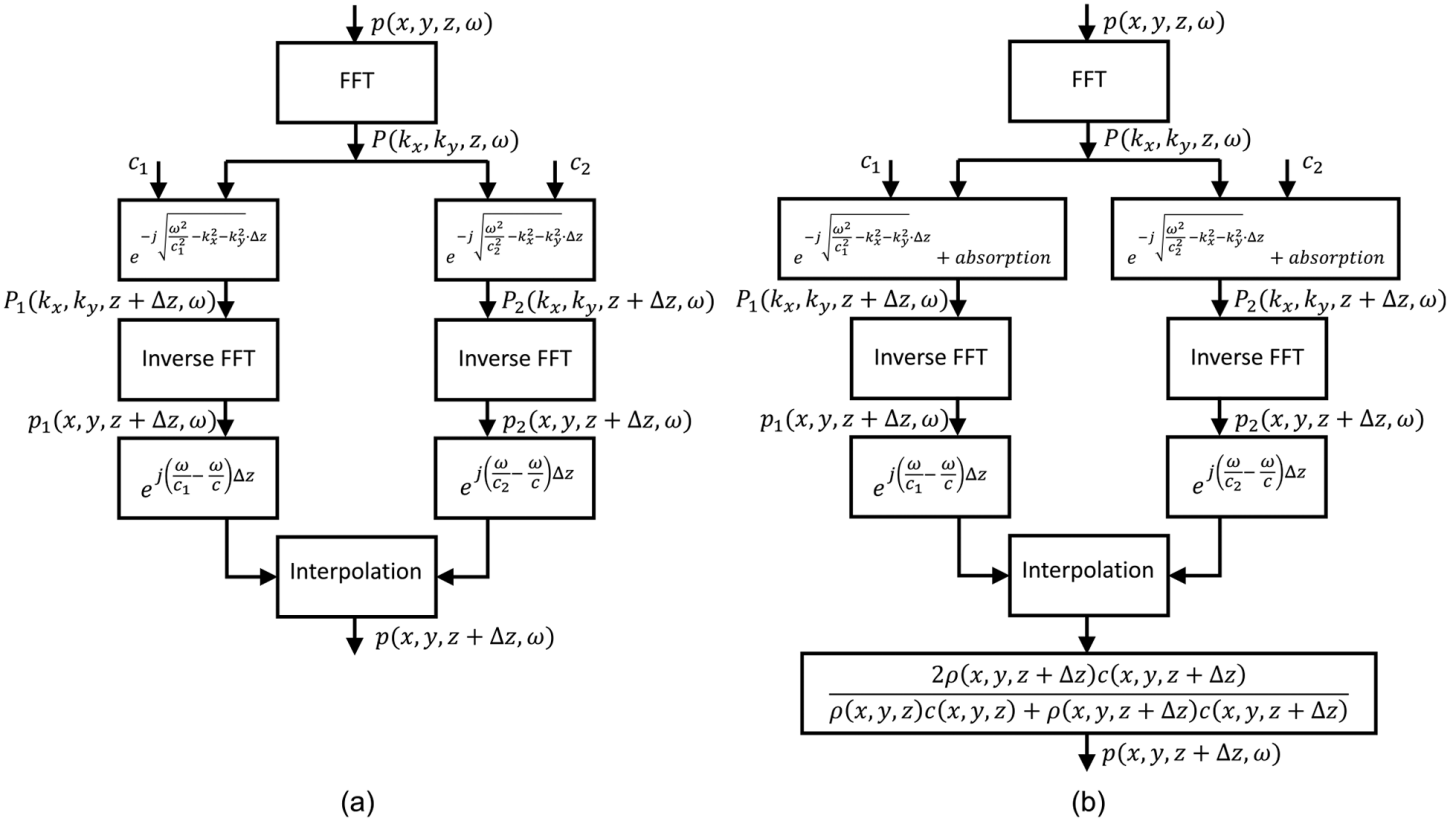
Flowchart highlighting the preexisting ESSF method (with the added interpolation step by Biondi [29]) in (a) and the modifications to it incorporated within mSOUND for this study in (b). Those modifications consist of the addition of absorption and the calculation of the transmission coefficient at the final step.

Fig. 1(a) details the steps comprising the ESSF method used in geophysical imaging. These steps consist of propagating forward with the ASA using two reference speeds of sound, generally chosen as the minimum and maximum speeds of sound (c_1_ and c_2_) over the medium. Then, a series of interpolation factors are evaluated to back out phase correction terms to account for the actual speed of sound at each (x, y) location between the two reference velocities [28]. This interpolation was not part of Kissinger’s original configuration of ESSF but was incorporated by Biondi in 2006 [29]. As a further extension to the original ESSF method, Fig. 1(b) highlights the approaches incorporated in mSOUND that account for absorption and transmission loss (due to reflection). The absorption follows the approach outlined in [30], wherein the governing equation is modified and Fourier transformed into a form akin to (2), in which the absorption is considered as part of the source term on the right-hand side. The solution is then derived from the 1D Green’s function. This approach allows for a spatially dependent absorption coefficient. The final step of Fig. 1(b) denotes calculation of the transmission coefficient under a plane wave and layered-medium approximation. The formula shown on the bottom of Fig. 1(b), strictly speaking, is only valid for normal incidence, and therefore presents a source of error in mSOUND introduced by this approximation. Of note, it is not until this step that density heterogeneities are accounted for. Because mSOUND primarily is focused on the more clinically relevant normal (or small angle) incidence, shear waves are not considered in the current model. Additionally, multiple reflections within the skull, between the inner and outer tables, are needed for accurate simulations. As such, the reflection coefficient is calculated from the transmission coefficient via the pressure continuity condition. There is a parameter within mSOUND to set the reflection order so that the user can determine how many internal reflections within the skull to account for, consisting of forward and backward reflections (even-order reflections correspond to one-way propagation in the +z-direction whereas oddorder reflections correspond to one-way propagation in the −z-direction). More details on how multiple reflections are modeled can be found in [30]. In summary, the current version of mSOUND can model diffraction, arbitrary absorption, strongly heterogeneous media in speed of sound, density, and absorption coefficient, scattering through an ad hoc approach of multiple reflections, all in the frequency domain without nonlinearity.

## III. METHODS

To demonstrate mSOUND’s effectiveness in clinical applications, models obtained from *in vivo* skulls were used for simulations. The protocol to obtain these *in vivo* skull scans was approved by the Institutional Review Board at the Washington University School of Medicine (Washington University in St. Louis, 201312136, 02/18/2019). All patient-identifying information was removed. Patient anonymity was preserved, and the principles of the Declaration of Helsinki were followed. All procedures followed the guidelines set forth by the Health Insurance Portability and Accountability Act Privacy Rule.

This study selected two patients diagnosed with glioblastoma between November 2014 and December 2023. CT images were acquired for each patient using a CT scanner (SOMATOM Confidence, Siemens, Munich, Germany & Brilliance 64, Phillips, Amsterdam, Netherlands) with the following scanning parameters: tube energy: 120 kVp, slice thickness: 1–1.5 mm, in-plane resolution: 0.5 – 0.98 mm. Skull A is of a 65-year-old female and skull B is of a 36-year-old male. The skull maps in HU were interpolated to a 0.25 mm discretization using the ‘nearest’ method in MATLAB so that abrupt discontinuities were preserved. Conversions from HU to speed of sound (c) and density (ρ) in (4) and (5) were made following the approach presented by Marsac et al. [27]:

(4) $$\rho =\rho_{min}+\left(\rho_{max}-\rho_{min}\right)\frac{HU-HU_{min}}{HU_{max}-HU_{min}}$$

(5) $$\text{c}={\text{c}}_{min}+\left(c_{max}-c_{min}\right)\frac{\rho -\rho_{min}}{\rho_{max}-\rho_{min}}$$

The minimum speed of sound and minimum density were taken to be that of water, namely 1500 m/s and 1000 kg/m^3^. The maximum density was taken to be 2100 kg/m^3^ following Aubry et al. in [23] whereas the maximum speed of sound was taken to be a variable parameter, to be discussed later. Meanwhile, a porosity-based approach presented by Constans et al. [31] was implemented to determine a spatially dependent absorption coefficient (α) in (6)–(8).

(6) $$\text{absorption}=\alpha f^{\gamma }$$

(7) $$\alpha =\alpha_{0}\sqrt{\Phi }$$

(8) $$\Phi =\frac{\rho_{max}-\rho }{\rho_{max}-\rho_{min}}$$

Equation (6) denotes a power law absorption model, where f is the frequency and γ, the power law exponent, is taken to be 2 throughout the domain, following [20]. Equations (7) and (8) are only applicable through the skull, where $\alpha_{0}=8\;\text{dB}/\text{cm}/{\text{MHz}}^{2}$ and Φ is the porosity [31]. Outside the skull, $\alpha =0.002\;\text{dB}/\text{cm}/{\text{MHz}}^{2}$, the value for water as found in [32].

Two skull segments were extracted from each subject, namely the top and back, resulting in four skull segments overall analyzed in this study. In seeking to isolate skull-induced effects, the skull segments were assumed to be submerged in water, ignoring the scalp, brain, and any other soft tissue. Because the patients had undergone previous treatments that resulted in bores through the skull that were filled with bone cement, skull segments were truncated and/or rotated slightly in order to avoid those sections. Bone cement has a significantly higher speed of sound and density compared to the skull, so their inclusion would have led to skewing the conversion from HU to acoustic variables to values lower than reality.

**FIGURE 2.**
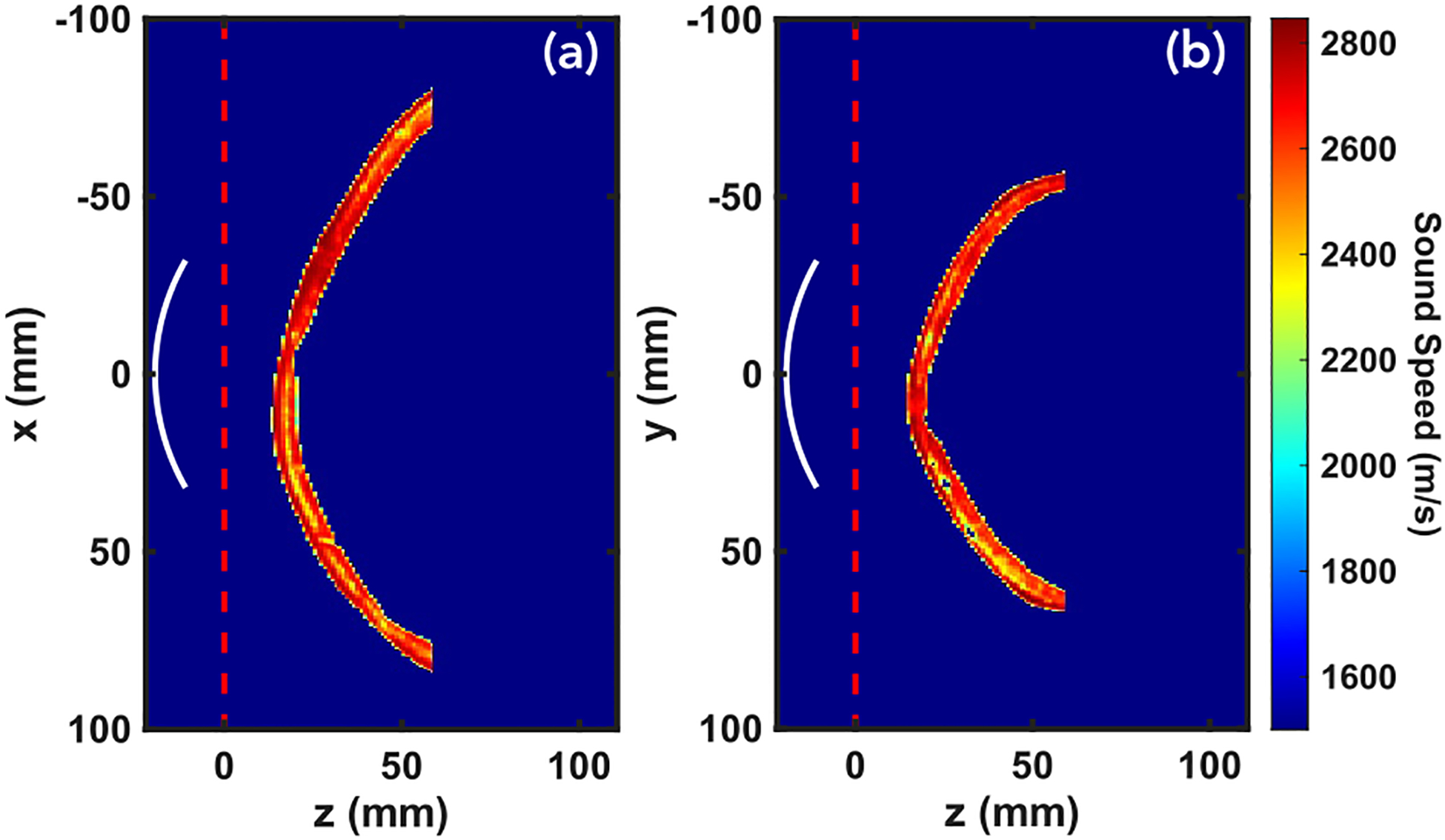
Relative position of the curved transducer to the top of skull A after being converted from HU to speed of sound, with a maximum of 2900 m/s. (a) depicts the x-z plane when y = 0 mm. Similarly, (b) depicts the y-z plane when x = 0 mm. The red dashed line at z = 0 mm corresponds to the transition from the near-field solved by FOCUS and the rest of the domain solved by mSOUND.

These submerged skull samples, along with the parameters outlined in the benchmark study [20], served as the medium for comparing mSOUND and k-Wave. k-Wave was chosen due to its high degree of accuracy and widespread use for 3D acoustic simulations. It should be noted that k-Wave’s underlying modeling equations are different than mSOUND’s. k-Wave solves a series of coupled first-order partial differential equations (PDE) whereas mSOUND solves the inhomogeneous Helmholtz equation in the frequency domain. For this study, a spherically curved transducer was considered, with a 64 mm radius of curvature and aperture diameter, the same as the one used in the previous intercomparison benchmark paper [20]. The frequency was 500 kHz with a pressure magnitude of 60 kPa at the transducer surface. Due to the current planar input constraints of mSOUND, another open-source wave solver FOCUS was used to evaluate the near-field of the curved transducer, which was subsequently saved over an x-y plane just past the transducer and treated as the initial condition in mSOUND to propagate the wave forward and through the skull. For all figures in this study, propagation is assumed in the z-direction, with z = 0 mm denoting the plane where computation was switched from the nearfield solved by FOCUS to the “skull domain” solved by mSOUND. All of this “skull domain” is considered to be filled with receivers for both mSOUND and k-Wave in order to compare the entirety of the pressure field of each. The setup highlighting the relative position of the transducer to the top of Skull A can be seen in Fig. 2. The dimensions of the total domain consisting of both the FOCUS and mSOUND sections were 280 mm ×280 mm ×120 mm with the first 19.5 mm belonging to the near-field solved by FOCUS. To avoid artificial lateral reflections inherent in angular spectrum approaches, a nonreflecting layer (NRL) built into mSOUND was employed, with parameters medium.NRL_gamma = 0.2 and medium.NRL_alpha = 0.3. The reflection order (for accounting for internal reflections within the skull) was set to be 4. Meanwhile, the dimensions of the k-Wave domain were 220 mm ×220 mm ×90 mm. The standard step size in all directions for both solvers was set to 0.25 mm following the interpolated HU map. For a sound speed of 1500 m/s and a 500 kHz source signal, this discretization corresponds to a points per wavelength (PPW) of 12. To analyze the effect of step size on computation time, two other discretizations were considered for both solvers, namely 0.5 mm and 0.2 mm corresponding to 6 and 15 PPW, respectively. Our computational resource constraints prevented even finer resolutions. It should be noted that for benchmark 7 (3D simulation of a skull segment submerged in water) in the intercomparison benchmark paper [20], wherein k-Wave was considered the ground truth, a PPW of 30 was used. For k-Wave’s time-domain simulation, a Courant–Friedrichs–Lewy (CFL) number of 0.1 was assigned resulting in a time step of approximately 16.7 ns. We also made use of k-Wave’s built-in perfectly-matched layer (PML) to eliminate artificial reflections in all directions. k-Wave’s function ‘getOptimalPMLSize’ was used to obtain PML width parameters that resulted in total grid point values that had the smallest prime factors in order to speed up FFT calculations. Following the parameters used in the previous intercomparison study, the ‘pml_alpha’ parameter was set to 2 Nepers per grid point [20]. The lateral dimensions of the mSOUND + FOCUS domain were wider to accommodate a wider NRL compared to k-Wave’s PML, necessary due to the inherent performance capabilities of the two mechanisms. PML’s generally require shorter thicknesses to achieve near-zero reflection, which k-Wave is able to make even shorter through its use of a staggered spatial grid [19]. In terms of boundary conditions, the Fast Nearfield Method (FNM) was the solver within FOCUS used for evaluating the nearfield of the curved transducer. It consists of a semi-analytical integral and accordingly no boundaries need to be explicitly defined. The plane it is propagated to is saved and used as the z = 0 source plane for mSOUND. To address the spatial aliasing error causing artificial reflections from the boundaries, mSOUND requires an NRL in the x and y directions. k-Wave, by contrast, makes use of a PML in all 3 directions. Thus, both solvers treat the domain as free-field and to do so, pad the boundaries with absorptive layers where necessary. Computation times were only evaluated over the respective software’s principal solver without pre- or post-processing considered (k-Wave’s “kspaceFirstOrder3DC.m” was chosen to make use of its more efficient C++ code). Simulations were all performed on a windows machine with 1012 GB of memory and an AMD EPYC 7702P 64-Core 3.2 GHz CPU.

To analyze the effects of maximum speed of sound on accuracy, three maximum speeds of sound were chosen: 2700, 2900 and 3100 m/s. 2900 m/s was the value used in [23] whereas 2700 and 3100 m/s were chosen by the authors as deviations from the central value. Six error metrics were evaluated, taken from the benchmark study, namely the relative $L^{2}$ and $L^{\infty }$ norms, focal (peak) pressure, and focal position metrics. The $L^{2}$ and $L^{\infty }$ norms were computed over a larger domain of dimensions 120 mm ×120 mm×192.5 mm as well as a smaller focal domain whose dimensions were determined to be where the pressure magnitude was −6 dB relative to the peak pressure in all directions (measured from k-Wave’s focal position). This smaller domain served to distinguish mSOUND’s accuracy around the region of interest near the focus from the more complex region within and in front of the skull. For each metric, data were averaged over the four skull samples, with the standard deviation evaluated, and then, we performed a series of statistical analyses. First, a one-way analysis of variance (ANOVA) was conducted for each error metric to assess whether the three maximum speeds of sound (2,700 m/s, 2,900 m/s, and 3,100 m/s) significantly influenced the errors across the four samples. The null hypothesis for each ANOVA assumed that the mean values of the error metric were equal across the maximum speeds of sound. A significance threshold of p <0.05 was used to determine whether the null hypothesis could be rejected, indicating that differences in mean error metrics were statistically significant.

For metrics where the ANOVA results were significant, Tukey’s Honestly Significant Difference (HSD) test was employed as a post-hoc analysis to identify specific pairwise differences between the speeds of sound. This test served to determine whether there was one speed of sound that was an outlier compared to the other two or whether the error value for each was distinct, indicating a trend. To further investigate the relationship between speed of sound and error metrics, Pearson’s correlation coefficient (r) was calculated for each metric. This coefficient quantified the strength and direction of an assumed linear relationship, with values ranging from −1 to 1. Positive values indicated a direct relationship, while negative values suggested an inverse relationship.

## IV. RESULTS

**FIGURE 3.**
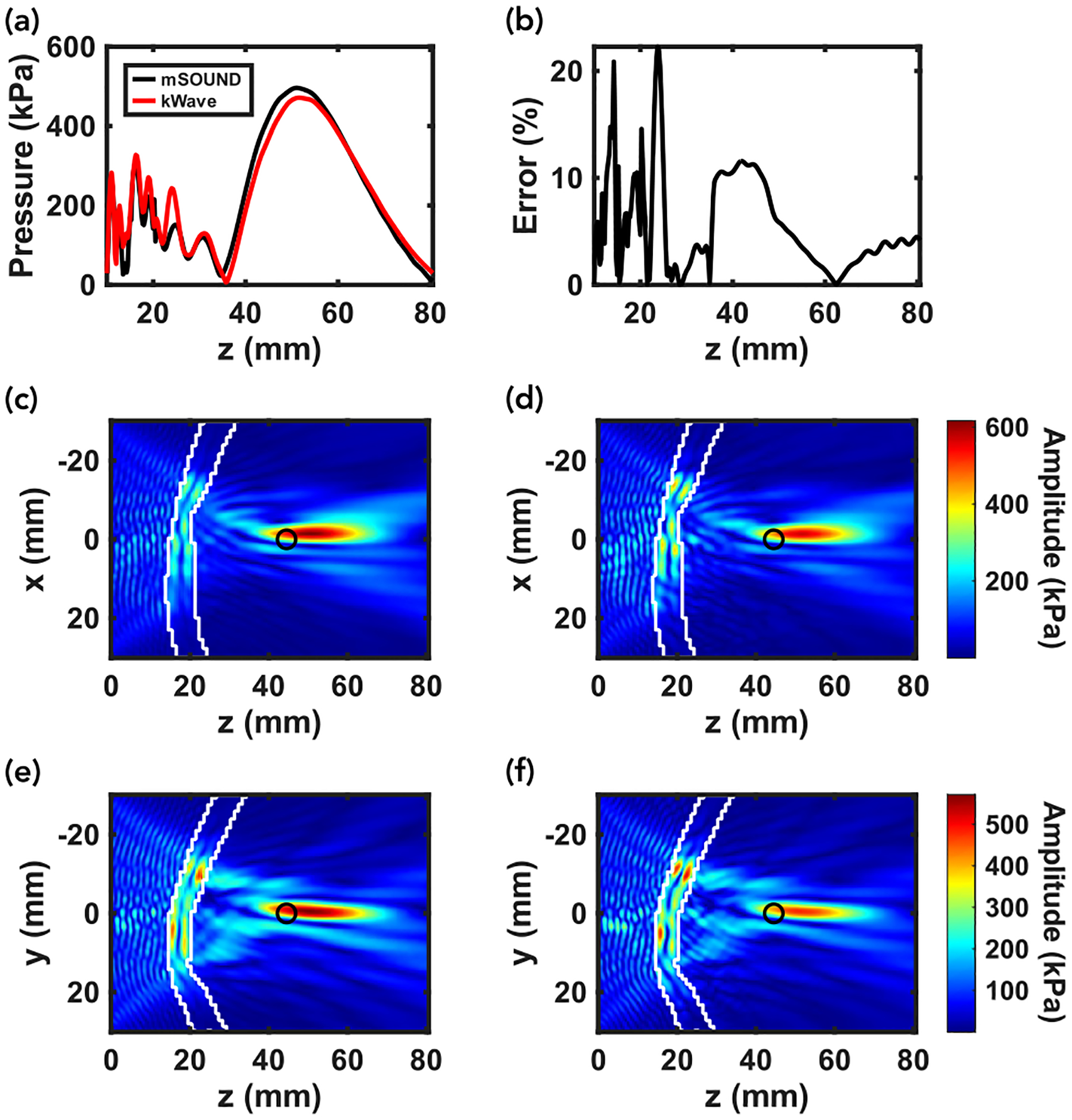
Results of simulating the acoustic field produced by the transducer-skull sample depicted in Fig. 2. (a) and (b) present the axial pressure distribution along the central z-axis and the percentage error over the same (measured relative to k-Wave at any particular point). (c) and (d) give the x-z planar pressure distribution for mSOUND and k-Wave, respectively, Similarly, (e) and (f) give the y-z planar distribution for mSOUND and k-Wave, respectively. Overlaid onto (c)–(f) are white lines representing the boundaries of the skull sample and black circles denoting the theoretical focal position absent the skull.

We begin with presenting the results in comparing k-Wave and mSOUND for the top of Skull A and a maximum speed of sound of 2900 m/s. Table 1 lists the previously described six error metrics as well as some additional quantitative metrics, including the overall maximum pressure, full width at half maximum (FWHM) in all three directions, focal volume, and focal shift. The focal volume was calculated by counting the number of voxels whose pressure exceeded half the maximum pressure near the focus (k-Wave in particular predicted high pressure values within the skull which were excluded) and multiplying that number by the product of the three spatial step sizes. The focal pressure error metric compares mSOUND’s maximum to k-Wave’s whereas the max pressure quantity is simply the maximum pressure of each pressure field in kPa. Finally, the focal shift is the distance from the theoretical focal position absent the skull to the simulated pressure field’s focal position whereas the focal position error metric compares mSOUND’s focal position relative to k-Wave’s. Fig. 3(a) and (b) show the pressure distributions along the z-axis obtained from the two solvers (where x,y=0). Characteristic of other skull samples, Fig. 3 highlights the trend of great variability of agreement between the solvers through the skull with a more consistent trend around the focus, with mSOUND predicting a somewhat higher peak focal pressure relative to k-Wave. Fig. 3(c)–(f) showcase two-dimensional pressure distributions with (c) and (e) representing mSOUND and (d) and (f) representing k-Wave. Of note is the excellent agreement in the focal area as well as the considerable pressure predicted in the outer and inner tables of the skull by both solvers. Particularly noticeable in Fig. 3(f), k-Wave predicts ‘bright spots’ of higher pressure trapped in the skull that mSOUND does not to the same extent.

**FIGURE 4.**
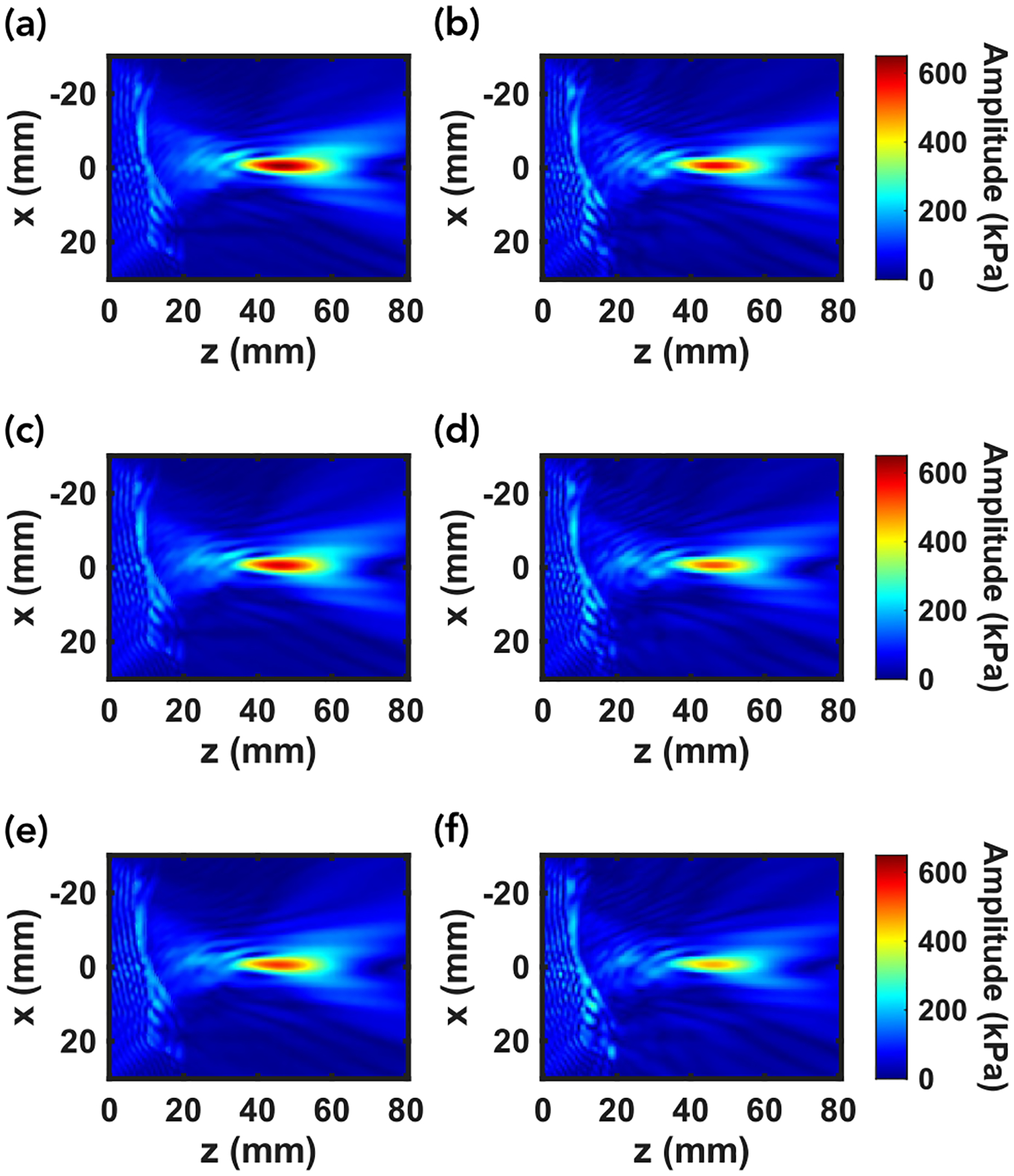
x-z planar results for simulating the acoustic field through the back of skull B. (a), (c), and (e) represent mSOUND while (b), (d), and (f) correspond to k-Wave. The top, middle, and bottom rows correspond to a maximum speed of sound of 2700, 2900, and 3100 m/s, respectively. The focal shape does not noticeably change with the maximum speed of sound however the peak pressure does decrease.

**FIGURE 5.**
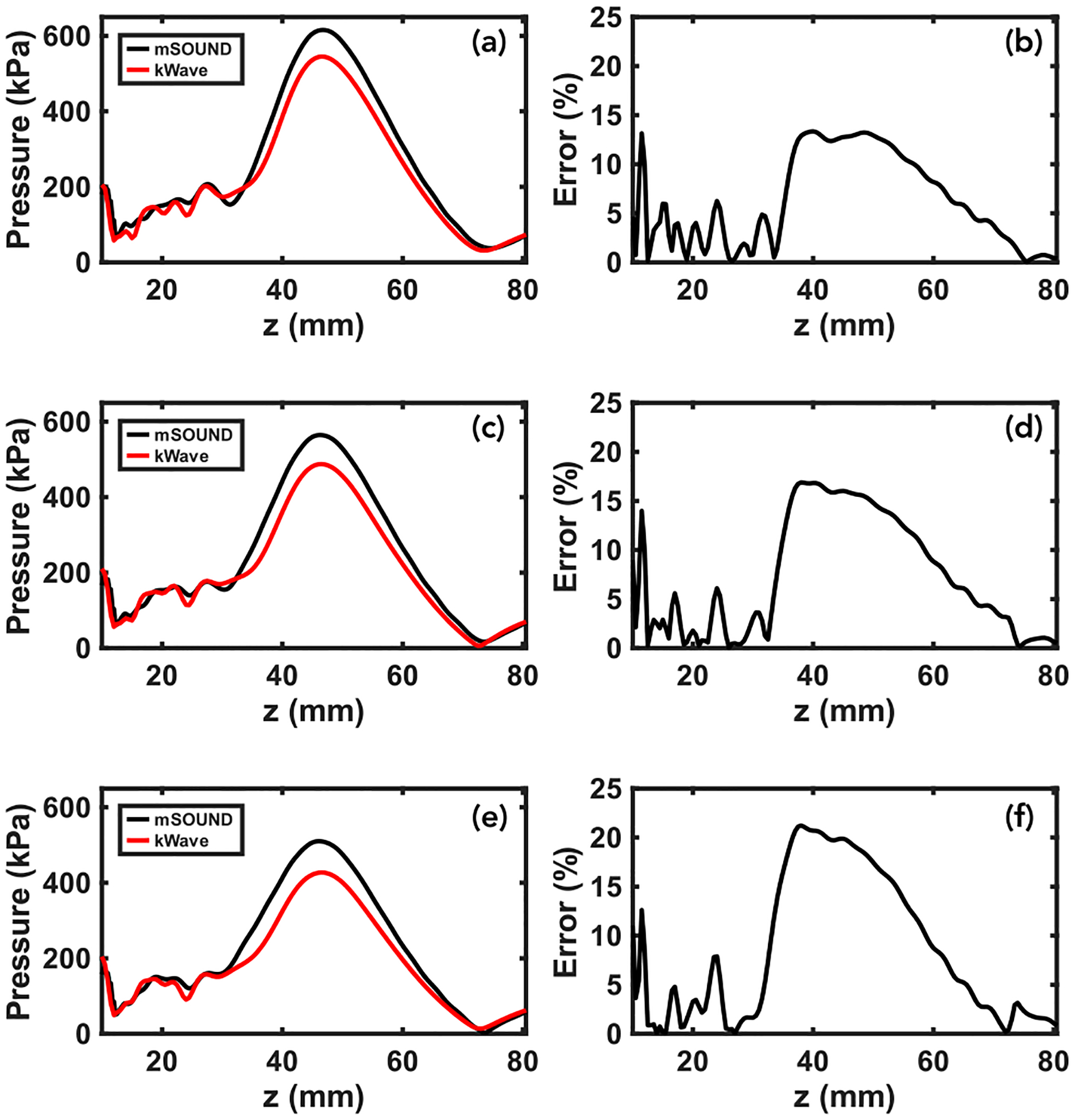
Axial pressure distribution results for simulating the acoustic field through the back of skull B. The top, middle, and bottom rows correspond to a maximum speed of sound of 2700, 2900, and 3100 m/s, respectively. Parts (b), (d), and (f) depict the percentage error for (a), (c), and (e), respectively. For this skull sample, the error over the z-axis is greatest around the focus, increasing as the maximum speed of sound increases.

Figs. 4 and 5 highlight the effects of changing the maximum speed of sound for another skull segment, namely the back of skull B. Notably, the shape and size of the focal region does not change noticeably as the maximum speed of sound changes. However, because the source HU were unchanged, extending the upper limit of the speed of sound to which the HU are mapped effectively increases the difference in acoustic impedance from voxel to voxel. This greater impedance mismatch leads to higher reflection coefficients and more acoustic energy reflected back towards the transducer as well as internally reflected within the skull. Consequently, both solvers predict a lower peak pressure as the maximum speed of sound increases. The implication is twofold: assuming an incorrect uniform maximum speed of sound across skulls will lead to noticeable errors in the predicted focal pressure, and skulls that are denser/have a higher speed of sound will result in a lower focal pressure.

In analyzing the effect of the varying maximum speed of sound on the relative performance of k-Wave and mSOUND, Figs. 5 (b), (d), and (f) offer some insight. The relative agreement before and through the skull changes but is sporadic and does not indicate any general trends. However, there is a noticeable trend in the error in the focal region. Specifically, the maximum focal percentage error grows from approximately 13.3% to 16.9% to 21.2% for 2700, 2900, and 3100 m/s, respectively. This trend indicates that the greater impedance mismatch resulting from a higher maximum speed of sound worsens mSOUND’s overestimation of the peak focal pressure.

**FIGURE 6.**
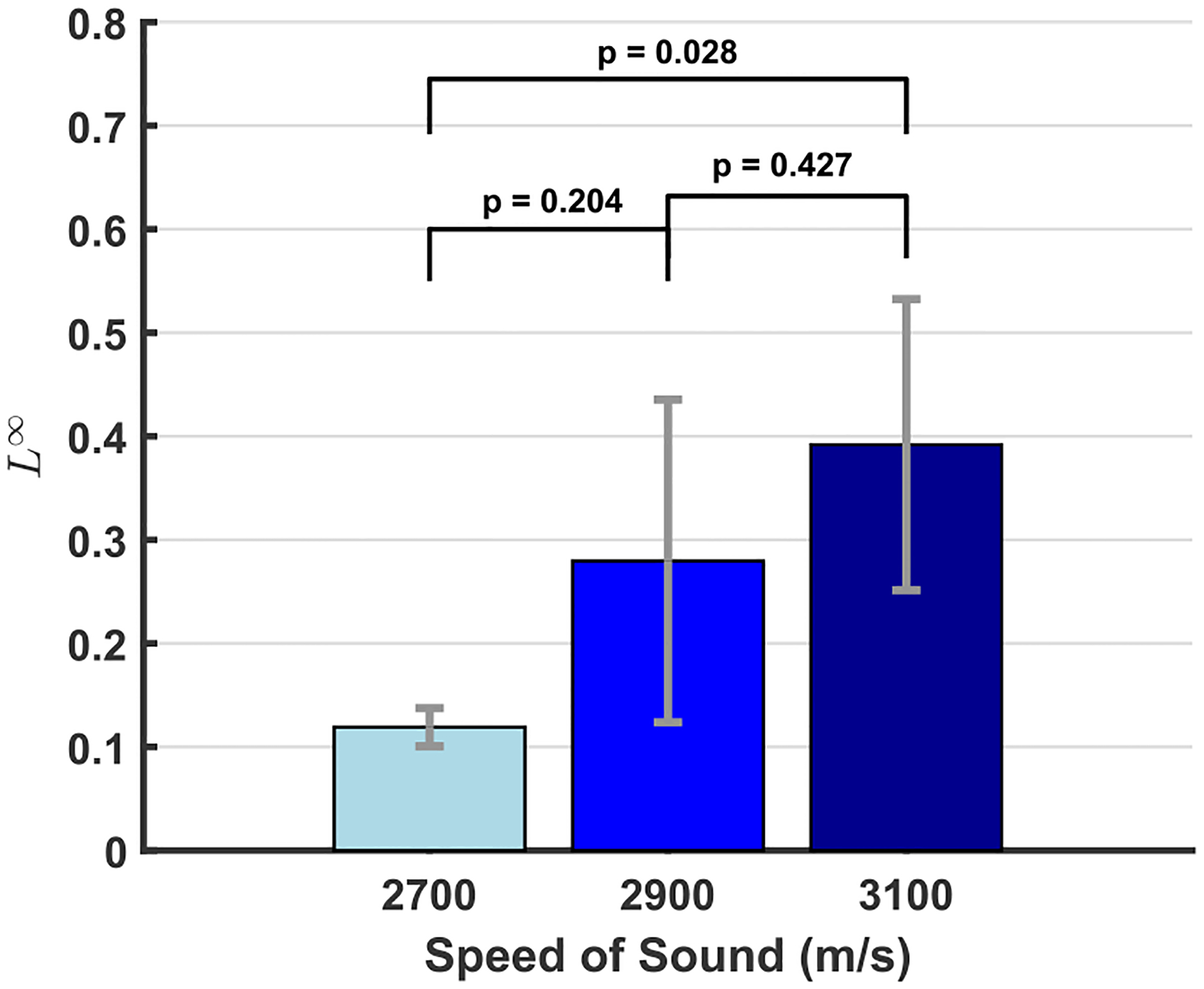
Result of the Tukey HSD test on the $L^{\infty }$ focal domain error metric, including standard deviation error bars. The p-values for each pairing are displayed over the respective bars, with only the p-value for the (2700 m/s, 3100 m/s) pair low enough to imply statistically significant differences between the groups.

**FIGURE 7.**
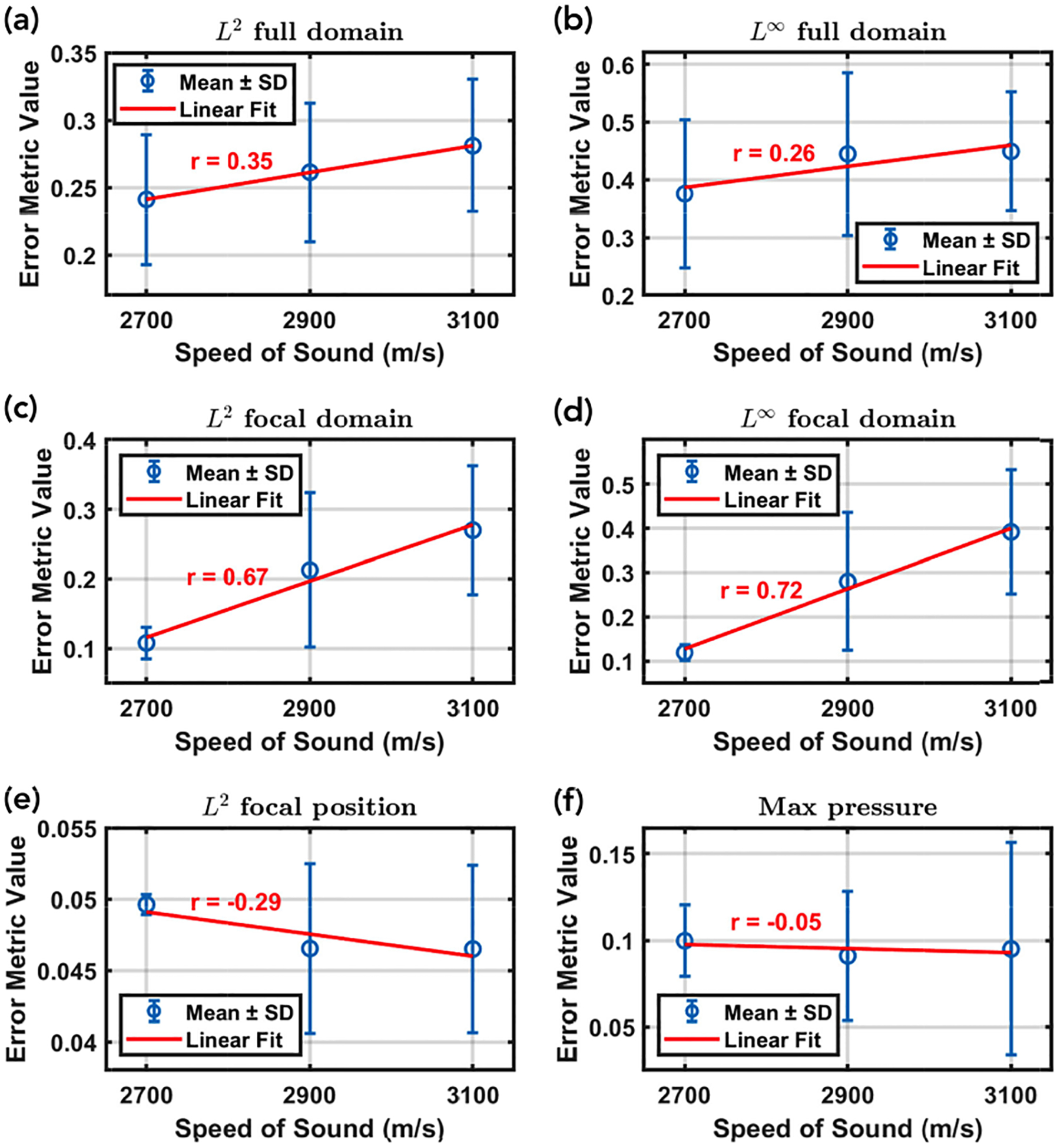
Effects of changing the maximum speed of sound on six different error metrics. The mean and standard deviation is calculated over all 4 skull samples with Pearson’s correlation coefficient given in red. By far, the two focal domain metrics exhibit the strongest trends of increasing error with increasing maximum speed of sound.

Of the six error metrics that were evaluated using the one-way ANOVA test, only the $L^{\infty }$ norm over the focal domain had a p-value less than 0.05 (0.033), although the $L^{2}$ norm over the focal domain was close, with a p-value of 0.065. Fig. 6 presents the results of the Tukey HSD post-hoc test for the focal domain $L^{\infty }$ norm, with only the results for the (2700 m/s, 3100 m/s) pair indicating statistically significant differences (p = 0.028, 95% confidence interval = [−0.513, −0.033]). However, there is a clear increasing visual trend, albeit with wide standard deviation bars. These trends are more explicitly visualized in Fig. 7, with the same $L^{\infty }$ and $L^{2}$ focal domain norms exhibiting the highest correlation coefficients.

Fig. 7 also details how the focal domain error is much less than the fuller domain error across the board. Furthermore, the error for the focal position is very low, indicating that mSOUND and k-Wave are in very close agreement for the location of the focus.

In terms of computation time, we found no meaningful differences across skull samples. For the top of Skull A, at the 0.25 mm spatial step size (12 PPW) used throughout this study, k-Wave’s kspaceFirstOrder3DC.m took 11 hrs 20 min 38.15 s while the FOCUS nearfield calculation took 4 min 47.10 s and the subsequent mSOUND calculation took 6 min 4.28 s. At a 0.5 mm spatial step size (6 PPW), k-Wave took 1 hr 46.48 s whereas FOCUS took 4 min 47.77 s and mSOUND took 36.45 s. Finally, at a 0.2 mm spatial step size (15 PPW, about the limit of our available memory), k-Wave took 30 hrs 6 min 36.71 s whereas FOCUS took 29 min 51.17 s and mSOUND took 6 min 28.55 s. While the difference in computation times is substantial, it should be noted that this was a monofrequency simulation, of which mSOUND is able to solve in the frequency domain whereas k-Wave is a time-domain solver, making this a comparison between a 3D and 4D simulation.

## V. DISCUSSION

In achieving computation times two orders of magnitude faster than k-wave while preserving comparable accuracy, particularly in the focal region, we have achieved the intended accuracy-efficiency balance. The accuracy varies considerably in the near-field and through the skull, in some cases (as in Fig. 5) demonstrating greater agreement than around the focal region. The greater agreement through the skull found in Fig. 5 compared to Fig. 3(a) and (b) may be due to the fact that the back of skull B used in Fig. 4 is about 3 mm thick through the z-axis whereas the top of skull A used in Fig. 3 is about 6 mm thick. Across skull samples, however, there is a consistent trend of mSOUND overestimating the peak focal pressure relative to k-Wave by anywhere from 6% to 21%. For all skull samples except the top of skull A, as the maximum speed of sound increases, which corresponds to a greater impedance mismatch voxel-to-voxel, the peak focal pressure error increases.

For this paper, k-Wave is considered as a benchmark solution, due to its well-documented accuracy [33]. Possible reasons for the discrepancy between mSOUND and k-Wave include numerical errors within each, the different model equations used, differences in the approach to reflection, and different approaches to accounting for diffraction effects through changing speeds of sound and density. To minimize numerical errors, we can further improve the spatial resolution, but we are currently limited by computational constraints. As mentioned previously, most simulations in this study were conducted at 12 PPW in water for both solvers whereas the most similar benchmark in the intercomparison study [20] was conducted at 30 PPW for k-Wave. However, the current simulation setup represents typical spatial and temporal resolutions achievable using standard computers commonly available in research laboratories.

As mentioned before, k-Wave is based on solving three coupled first-order PDEs while mSOUND fundamentally solves the inhomogeneous Helmholtz equation. In addition, unlike k-Wave, which is a full-wave solver, mSOUND is inherently a one-way solver and so can only account for internal reflections and transmission loss within the skull in an *ad hoc* manner. One potential approach to improve the transmission loss in mSOUND is to adopt the approach presented in [34], wherein the Lippmann-Schwinger integral equation was solved to obtain a general expression for the transmission coefficient in a medium with constant density but inhomogeneous speed of sound. As noted before, the ‘bright spots’ within the skull that k-Wave predicts to a greater extent than mSOUND indicate that more acoustical energy is being trapped within the skull than mSOUND is able to capture. With an improved transmission coefficient and subsequent reflection model, we may be able to better predict how much energy is trapped within the skull and consequently reduce the overestimation error of the peak focal pressure.

Beyond improving the accuracy of mSOUND, we also plan to experimentally validate mSOUND using *ex vivo* skulls [35], during which we can do an independent analysis of maximum speed of sound and density parameters to optimize agreement. Additionally, we can further improve mSOUND’s computational savings by incorporating GPU acceleration. We can also add greater functionality within mSOUND itself to accommodate nonplanar sources by implementing the Rayleigh integral, for instance. Although this work followed the monofrequency, linear acoustics assumption made in the intercomparison study [20], nonlinear effects, especially at high enough amplitudes, need to be considered for accurate tFUS modeling [36], [37]. mSOUND already includes a transient nonlinear solver [38], but it has not yet been updated to also accommodate strong heterogeneities, which we can investigate in the future. Furthermore, the spatially varying absorption coefficient model presented in this study follows a single scalar power-law model which may not be the most accurate for broadband applications, of which the simulations presented here are not. mSOUND has an inherent advantage over k-Wave in this regard, in that being a frequency-domain solver allows for an arbitrary dispersion and frequency-dependent absorption model. This advantage can be more fully realized in future broadband simulations upon mSOUND’s transient solver being updated to incorporate the strong heterogeneity capability presented here. Additionally, with the increasing use of deep learning for tFUS modeling [39], mSOUND’s computational speed offers a far faster alternative than k-Wave and other full wave solvers to generate a vast training dataset. Finally, we can incorporate mSOUND into the clinical pipeline for phase correction, allowing for transcranial beam focusing.

## VI. CONCLUSION

In this paper, we present a solver not yet reported within the open-source MATLAB toolbox mSOUND for acoustic simulations involving strongly heterogeneous media. Particularly for this study, we focus on mSOUND’s performance simulating tFUS, comparing it against the well-established full-wave solver k-Wave. We compared k-Wave and mSOUND over four separate skull segments and three maximum speed of sounds, a parameter assigned by the user in converting from HU to the speed of sound.

The combined computation time of mSOUND and FOCUS (used to solve the near-field of the focused transducer) was approximately 1.6–8.9% that of k-Wave (depending on the step size), highlighting substantial computational savings allowing for greater feasibility for modeling tFUS for clinicians with limited computational resources or who have applications of an iterative or time-sensitive nature. mSOUND’s accuracy relative to k-Wave varies considerably outside and through the skull, largely depending on the complexity and thickness of the skull itself. In the focal region, mSOUND and k-Wave have excellent agreement in the position and shape of the focus. mSOUND consistently predicts a higher focal pressure of 6–21% greater than k-Wave’s, which generally increases as the maximum speed of sound parameter increases. In the future, we plan to improve mSOUND’s accuracy without sacrificing computational efficiency by improving our transmission loss and reflection model as well as experimentally validate mSOUND and incorporate it into clinical applications such as BBB opening and neuromodulation.

## Figures and Tables

**TABLE 1.** K-Wave vs. mSOUND, Top of Skull A, Max Speed of Sound = 2900 m/s.

Metrics	k-Wave	mSOUND (+FOCUS)
Step Size (mm)	0.25	0.25
Computational Time	11 hrs 20 min 38.15 s	10 min 51.38 s
Max Pressure (kPa)	571.4	619.1
FWHMx (mm)	4.5	4.5
FWHMy (mm)	4.25	4.5
FWHMz (mm)	27.5	28
Focal Volume (mm^3^)	297.9	315.2
Focal Shift (mm)	7.3	6.1
$L^{2}$ Focal Domain	0.111	
$L^{2}$ Full Domain	0.238	
$L^{\infty }$ Focal Domain	0.157	
$L^{\infty }$ Full Domain	0.373	
Focal Pressure	0.0835	
Focal Position	0.0013	

The bottom six error metrics consider k-Wave as the ground truth to which mSOUND is compared. Note that the ‘Max Pressure’ quantity is distinct from the ‘Focal Pressure’ error metric, as is ‘Focal Shift’ from ‘Focal Position.’ The computational time for the second column consists of the combined time of both the near-field solved by FOCUS and the remaining domain solved by mSOUND.
